# Decoding the Transcriptional Complexity of the Human *BRCA2* DNA Repair Gene Using Hybrid-seq

**DOI:** 10.1007/s10528-025-11180-6

**Published:** 2025-07-10

**Authors:** Panagiotis G. Adamopoulos, Michaela A. Boti, Konstantina Athanasopoulou, Panagiotis Tsiakanikas, Glykeria N. Daneva, Andreas Scorilas

**Affiliations:** Department of Biochemistry and Molecular Biology, Faculty of Biology, National and Kapodistrian University of Athens, Panepistimiopolis, 15701 Athens, Greece

**Keywords:** Alternative splicing, Nanopore sequencing, Gynecological cancers, Transcriptomics, BRCA2 DNA repair associated gene

## Abstract

BRCA2 plays a pivotal role in DNA repair and tumor suppression, with its dysregulation linked to breast and gynecological cancers. Despite the importance of BRCA2, its transcriptional complexity remains poorly understood due to the gene's size and intricate alternative splicing patterns. This study aims to comprehensively characterize the BRCA2 transcriptional landscape in breast, ovarian, and cervical cancers using a hybrid sequencing approach. A novel hybrid-seq method combining long-read nanopore sequencing and short-read NGS was applied to analyze BRCA2 transcripts from cancerous cell lines. Expression patterns were evaluated using the transcript-per-million (TPM) normalization method, and open reading frames (ORFs) of the identified transcripts were in silico characterized. Sequencing analysis led to the identification of 50 novel splice variants (*BRCA2* sv.7–sv.56), expanding the known transcript repertoire of *BRCA2* gene. Notably, transcript variants sv.9, sv.15, and sv.49 exhibited significant expression in breast and ovarian cancers, while others, such as sv.29 and sv.40, were specific to individual cancer types. Five cryptic exons (N1–N5) were unveiled, contributing to 10 unique splice variants. In silico analysis revealed that 19 novel transcripts retained coding potential, with some encoding BRCA2 isoforms harboring key functional domains. The identification of novel BRCA2 transcripts underscores the complexity of its regulation in cancer. These findings provide insights into the gene's potential role in tumorigenesis and highlight candidates for targeted therapies and diagnostic biomarkers.

**Supplementary Information:**

The online version contains supplementary material available at 10.1007/s10528-025-11180-6.

## Introduction

Acknowledged as the steppingstone to precursor messenger RNA (pre-mRNA) maturation, RNA splicing constitutes an intricate post-transcriptional regulatory mechanism of gene expression, as well as one of the prime contributors to the staggering increase in the transcriptomic and proteomic complexity of eukaryotic organisms. In specific, via a finely tuned transesterification process executed by an enormous ribonucleoprotein complex known as the spliceosome, introns become excised from the pre-mRNA, while exons are joined together to consequently give rise to the mature RNA transcript. Interestingly, a combination of *cis*- and *trans*- regulatory elements tightly coordinates exon selection and determines the sequences to be included in the final mRNA product, hence increasing the number of the possible differentially spliced variants and resultant protein isoforms arising from a single gene—a mechanism known as alternative splicing (AS) (Chen and Manley 2009; Lee and Rio 2015; Marasco and Kornblihtt 2023). Evidently, alterations in the open reading frame (ORF) can determine in varied ways the “fate” of RNA transcripts, by either subjecting them to nonsense-mediated decay (NMD) or rendering them non-coding—with potential regulatory function—or even, on the occasion that the mRNAs preserve their coding capacity, by resulting in the modification of the generated protein’s subcellular localization, stability, binding properties and/or catalytic activity (Stamm et al. 2005).

Previous studies have already validated the complicity of Breast Cancer gene 2 (*BRCA2*) in various cancer types, highlighting its key role in tumor development and progression, especially in breast and ovarian cancer (Chakraborty et al. 2020; Junejo and AlKhateeb 2020; Saleem et al. 2020). Upon its identification in 1995, *BRCA2* was recognized as a tumor suppressor gene, with its corresponding protein to be involved in the repair of damaged DNA, resulting in the maintenance of genome stability, and participating in cell growth regulation. Briefly, in response to DNA double-strand breaks (DBS), special sensor molecules detect DNA damage and provoke the mediators to recruit and/or activate the effector molecules, so the latter repair the breaks. After the sensors’ action, BRCA2 which functions as a mediator molecule, interacts with BRCA1 and PALB2 (Gudmundsdottir and Ashworth 2006; Simhadri et al. 2019), forming a complex responsible for the recruitment of the effector RAD51 (Yuan et al. 1999; Yang et al. 2002; O'Donovan and Livingston 2010). Subsequently, RAD51 mediates DNA repair via the homologous recombination (HR) pathway, reinstating the occurred damage (Moynahan et al. 2001; Powell et al. 2002; Foo and Xia 2022). Based on its function, BRCA2 is considered to be a vital protein for the maintenance of cells’ physiological state and, thus, alterations in the corresponding gene can easily lead to cell cycle dysregulation and abnormal cell proliferation (Tian et al. 2005).

Inherited mutations in *BRCA2* are strongly linked to breast and ovarian cancer development, increasing the lifetime risk of these cancers in *BRCA2* mutation carriers (Consortium 1999; Antoniou et al. 2008). Owing to its involvement in hereditary breast and ovarian cancer, individuals with positive family history are checked for harmful *BRCA2* variants. The existence of *BRCA2* cancer-related variants, as well as the necessity of evaluating this gene’s mutational profile, reflects *BRCA2* significance in breast and ovarian cancer development and progression (Antoniou et al. 2008; Odemis et al. 2022). Of note, previous studies have already demonstrated that mutations in this gene affect splicing, generating variations in splice sites and leading to the production of pathogenic alternative *BRCA2* transcripts (Fraile-Bethencourt et al. 2019; Jasiak et al. 2023). The potential translation of the alternative transcripts that retain ORFs may generate truncated, non-functional protein isoforms that are associated with breast and ovarian cancer (Whiley et al. 2014; Gambino et al. 2015). Moreover, *BRCA2* is one of the most common genes found altered in prostate cancer, confirming its complicity in the particular disease and extending its role beyond ovarian and breast cancers (Consortium 1999; Oh et al. 2019; Junejo and AlKhateeb 2020; Hofstad et al. 2022; Saunders et al. 2024). Previous works have associated the impaired DNA damage repair mechanisms in prostate cancer with *BRCA2* deficiencies, reporting that mutations in this gene are linked to rapid progression of the disease and poor survival outcomes (Narod et al. 2008; Gallagher et al. 2010; Thorne et al. 2011; Castro et al. 2013).

*BRCA2* is located on chromosome 13q and consists of 27 exons, with exon 11 being the larger among the known exons. *BRCA2* encodes a tumor suppressor protein consisting of 3,418 amino acids which is characterized by the presence of four well-characterized distinct domains: a PALB-2 binding domain (BD), a RAD51-binding domain including eight BRC repeats, a DNA-binding domains (DBD), and a C-terminal RAD51-binding domain (TR2) (Le et al. 2021; Kwon et al. 2023). More precisely, the N-terminus contains two protein-interaction sides, one for PALB2 and one for EMSY, while the binding of PALB2 in BRCA2 N-terminus assists the recruitment of RAD51 to the damaged DNA. The core domain of BRCA2 consists of eight BRC repeats, encoded by exon 11, which constitute the primary interaction sites of RAD51 (Bignell et al. 1997; Wong et al. 1997; Chen et al. 1998). DBD harbors one helical region and three Oligonucleotide Binding (OB) folds and has specificity for ssDNA. The C-terminus contains two nuclear localization signals (NLS) and an additional interaction site for RAD51, while the particular region is equivalent to exon 27 (Esashi et al. 2007; Andreassen et al. 2021).

Alternative splicing mechanism has resulted in the generation of diverse BRCA2 transcript variants, the majority of which encode distinct protein isoforms. To date, five BRCA2 transcript variants have been annotated besides the main mRNA, namely NM_001406720.1, NM_001406719.1, NM_001406721.1, NM_001406722.1 and NR_176251.1. Compared to transcript variant 1 (NM_000059.4), variant 2 (NM_001406720.1) features a truncated exon 23, generating an alternative exon (23alt) which has a length of 113 bases. Transcript variant 3 (NM_001406719.1) lacks exon 12, while transcript variant 4 (NM_001406721.1) omits exon 11. Following, transcript variant 5 (NM_001406722.1) excludes exons 2, 10, and 11 from its sequence, and variant 6 (NR_176251.1) retains an intronic sequence of 64 bases from the corresponding pre-mRNA, resulting in a transcript with 28 exons (Supplementary Fig. 1). Except for variant 6, the rest of the transcripts described are coding, producing alternative protein isoforms that exhibit variations in both structure and function. The diversity of BRCA2 transcripts and their encoded isoforms plays a crucial role in regulating the protein's interaction with other DNA repair factors, its stability, and its ability to maintain genomic integrity. As one of the key players in homologous recombination repair, the function of BRCA2 can be significantly influenced by these isoforms, particularly in the context of cancer predisposition and therapy resistance.

Although the functional importance of BRCA2 in genomic stability and its association with cancer risk is well-established, comprehensive investigations of its transcriptional landscape remain scarce. Prior studies have predominantly focused on mutational profiling and its impact on protein function, with limited exploration of the gene's splicing diversity and transcriptional regulation. On that account, the transcriptional landscape of *BRCA2* is being poorly investigated, a fact that sets its study as a priority.

In the present study, to comprehensively characterize the transcriptional complexity of the human *BRCA2* gene in breast and gynecological malignancies, we employed a targeted hybrid sequencing (Hybrid-Seq) approach that combines both long-read nanopore sequencing (Oxford Nanopore Technologies Ltd., ONT, Oxford, UK) and short-read next-generation sequencing (NGS), using gene-specific primers for *BRCA2* gene. This integrative approach enabled the polishing of the error-prone nanopore sequencing reads, thereby leading to the generation of highly accurate long sequencing reads that were analyzed for the identification of full-length *BRCA2* transcripts (Supplementary Fig. 2). By implementing this *in-house* developed targeted DNA-seq approach, we successfully detected and characterized a wide spectrum of *BRCA2* splice variants (*BRCA2* sv.7 – sv.46), evaluated their expression levels across distinct cancer types, and provided novel insights into the gene’s dysregulation and potential functional relevance under cancer conditions.

## Methods

### Biological material

For the implementation of the present study, 11 human cancerous cell lines were utilized, as follows: MCF-7, SK-BR-3, BT-20, MDA-MB-231, MDA-MB-468, BT-474 (breast/ductal adenocarcinoma), OVCAR-3, SK-OV-3, ES-2, MDAH-2774 (ovarian cancer), HeLa (cervical cancer). All cell lines were cultured according to the guidelines of the American Type Culture Collection (ATCC). Upon thawing, all cell lines underwent at least three passages before being harvested for downstream experimental steps.

### Total RNA extraction, poly(A) selection and reverse transcription

Total RNA extraction was carried out from confluent T75 flasks (~ 5–7 × 10⁶ cells). Total RNA was isolated from each cell line using TRIzol Reagent (Ambion™, Thermo Fisher Scientific Inc., Waltham, MA, USA). RNA purity and concentration were determined spectrophotometrically by measuring absorbance at 260 and 280 nm, using a BioSpec-nano Micro-volume UV–Vis Spectrophotometer (Shimadzu, Kyoto, Japan). Subsequently, mRNA enrichment from 5 μg of each total RNA sample was achieved using the NEBNext® Poly(A) mRNA Magnetic Isolation Module (New England Biolabs, Inc.).

Reverse transcription (RT) was performed in 20 μl reaction volumes using 100 ng of mRNA from each cell line, oligo-dT_20_ as the primer, and Maxima H Minus Reverse Transcriptase (Invitrogen™, Thermo Fisher Scientific Inc.). Briefly, each initial cDNA synthesis mixture contained 12.5 μl poly(A) + RNA and 1 μl oligo-dT_20_ (10 μM), which was incubated at 65 °C for 5 min in a Veriti 96-Well Fast Thermal Cycler (Applied Biosystems™) with a heated lid. Subsequently, the cDNA synthesis mixture was completed by adding 4 μl of 5X RT Buffer, 1 μl of dNTP mix (10 mM each), 0.5 μl of RNaseOUT inhibitor (20 U; Invitrogen™, Thermo Fisher Scientific Inc.), and 1 μl of Maxima H Minus Reverse Transcriptase (200 U; Invitrogen™, Thermo Fisher Scientific Inc.). First-strand cDNA synthesis was conducted by incubating the reaction mixture at 50 °C for 30 min, followed by heat inactivation at 85 °C for 5 min. The quality of the synthesized cDNA was verified by amplifying the housekeeping gene *GAPDH* (glyceraldehyde 3-phosphate dehydrogenase). Finally, the cDNA samples were pooled equimolarly based on tissue of origin or cancer type (breast, ovarian and cervical cancers) to form 3 distinct cDNA pools, which were subsequently used as templates in downstream PCR-based assays.

### Amplification of BRCA2 splice variants

A touchdown PCR assay was developed and optimized using two gene-specific primers (GSPs) to selectively amplify *BRCA2* transcripts. The forward GSP (F: 5΄– AGAAGCGTGAGGGGACAGAT –3΄) was designed to anneal near the ATG start codon, while the reverse GSP (R: 5΄– TGGGAGCAGTCCTAGTGGAT –3΄) targeted the last annotated exon of the main mRNA, *BRCA2* v.1.

The PCR reaction mixture (50 μl) included 5 μl of 10X LA PCR Buffer II (with Mg^2^⁺), 8 μl of dNTP mix (2.5 mM each), 1 μl of each GSP (10 μM), 0.5 μl (2.5 U) of TaKaRa LA Taq polymerase, and sterile distilled water. Amplification was performed in a Veriti 96-Well Fast Thermal Cycler (Applied Biosystems™) with an initial denaturation at 94 °C for 3 min, followed by 35 cycles of 94 °C for 30 s, 65 °C (auto-ΔTa: − 0.3 °C/cycle) for 30 s, and 72 °C for 12 min, and concluding with a final extension at 72 °C for 15 min. PCR specificity and yield were assessed by agarose gel electrophoresis. Amplicons were purified using the NucleoSpin® Gel and PCR Clean-up kit (Macherey–Nagel GmbH & Co. KG, Düren, Germany) and quantified using the Qubit® DNA HS Assay Kit (Invitrogen™, Thermo Fisher Scientific Inc.).

### Targeted Hybrid-seq approach

A total of 1 μg of each purified amplicon was used to prepare seven DNA-seq barcoded libraries for nanopore sequencing. These libraries represented amplified BRCA2 mRNAs from breast, ovarian, and cervical cancers. Library construction was performed using the Ligation Sequencing Kit (SQK-LSK109, ONT) and the Native Barcoding Expansion Kit 1–12 (EXP-NBD104, ONT), following the manufacturer’s instructions. During preparation, end repair was carried out with the NEBNext® Ultra™ II End Repair/dA-Tailing Module (New England Biolabs, Inc.), adapter ligation was facilitated by Quick T4 Ligase (New England Biolabs, Inc.), whereas all clean-up steps utilized Agencourt AMPure XP beads (Beckman Coulter, Brea, CA, USA). Equimolar amounts of each barcoded library were pooled to create a final DNA-seq library, which was loaded onto a FLO-MIN106D flow cell with R9.4.1 chemistry. Nanopore sequencing was conducted on a MinION Mk1C sequencer (Oxford Nanopore Technologies Ltd., ONT, Oxford, UK).

For the Hybrid-seq approach, the same PCR products were used as templates for constructing DNA-seq libraries for next-generation sequencing. Library preparation was performed with the Ion Xpress™ Plus Fragment Library Kit (Ion Torrent™, Thermo Fisher Scientific Inc.) and included an initial enzymatic fragmentation step, adapter ligation with nick-repair, and purification of ligated DNA. A bead-based size selection was then conducted using the KAPA Pure Beads (Kapa Biosystems Inc.) at the recommended dsDNA:bead ratio, aiming for ~ 400 bp fragments. Library quantification was performed on an ABI 7500 Fast Real-Time PCR System (Applied Biosystems™) using the Ion Library TaqMan™ Quantitation Kit (Ion Torrent™). Finally, template preparation and enrichment were completed with the Ion PGM™ Hi-Q™ View OT2 kit (Ion Torrent™), and sequencing was conducted on the Ion Personal Genome Machine™ (PGM™) platform using the Ion PGM™ Hi-Q™ View Sequencing Kit.

### Bioinformatics analysis

Basecalling, demultiplexing, adapter trimming, and quality control (QC) analysis of the raw nanopore sequencing datasets were performed using Guppy and NanoPlot (De Coster et al. 2018) (Supplementary Data). Following analysis, reads were categorized into “pass” and “fail” folders based on quality scores, with only reads from the “pass” folder retained for investigating *BRCA2* transcriptional profile and identifying potential novel transcripts. Subsequently, a long-read polishing step was conducted using hybrid error-correction algorithms that integrated both the nanopore and short-read NGS datasets, generating highly accurate long reads for downstream bioinformatics analysis (Broseus et al. 2020). Polished long reads from each barcoded nanopore library were aligned to the human reference genome (GRCh38) using minimap2 (Li 2018), while read mapping was visualized using Integrative Genomics Viewer (IGV) (Robinson et al. 2011).

### Expression analysis of the identified BRCA2 splice variants

Demultiplexing of the polished nanopore sequencing reads enabled the identification of *BRCA2* transcript variants present in each barcoded library, along with the evaluation of their expression levels. For estimating the abundance of each *BRCA2* transcript in each library the transcripts per million (TPM) method was employed, while only sequencing reads covering the entire coding sequence—from the translation initiation site to the last exon—were included. Reads from random fragments generated during library preparation were excluded, as they did not represent unique variants. Identification of *BRCA2* splice variants in each dataset was achieved using the *in-house* developed tool ASDT (Adamopoulos et al. 2018). Additionally, specialized long-read algorithms for alternative isoform quantification were applied to estimate the abundance of each *BRCA2* splice variant in each barcoded library (Shumate et al. 2022).

### ORF characterization in the identified BRCA2 transcripts

Following the characterization of *BRCA2* transcriptional profile, an in silico ORF analysis was performed for each identified mRNA transcript. All novel *BRCA2* splice variants described in the present study were investigated for their coding capacity, using the ExPASy translate tool. In detail, the identified nucleotide sequence of each novel transcript was used to identify potential ORFs originating from the annotated initiation site (ATG) of the main mRNA BRCA2 v.1. Each ORF was subsequently evaluated according to the mammalian NMD rule and analyzed for the presence of conserved BRCA2 domains. Based on the NMD rule, transcripts with ORFs containing a termination codon located more than 50–55 nucleotides upstream of the last exon junction are likely to undergo NMD. Finally, Gene Ontology (GO) terms with significant prediction scores were assigned to each putative BRCA2 isoform using DeepGOWeb (Kulmanov et al. 2021).

## Results

### Long-read sequencing unveils novel BRCA2 mRNAs in breast and gynecological cancers

**Fig. 1:**
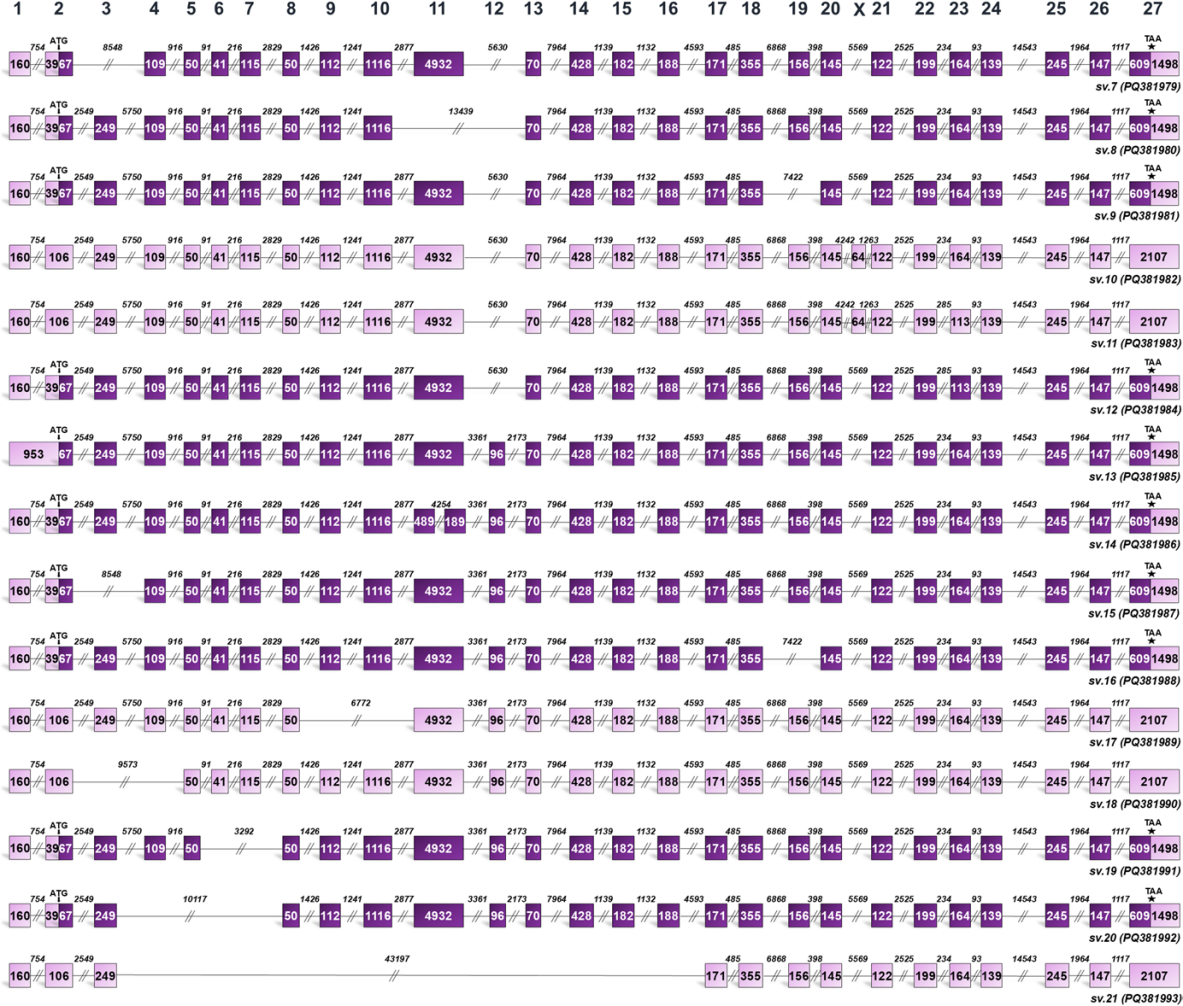
Structural illustration of novel *BRCA2* splice variants expressed in more than one cancer type investigated (*BRCA2* sv.7 – sv.21). Exons are exhibited as boxes and introns as lines. The numbers inside the boxes and above the lines correspond to the exon and intron lengths, accordingly. Dark purple boxes represent the coding region of the mRNAs that utilize the annotated initiation site of exon 2 and are predicted as protein-coding, while light purple is used to demonstrate non-coding RNAs with PTCs as well as untranslated regions

**Fig. 2:**
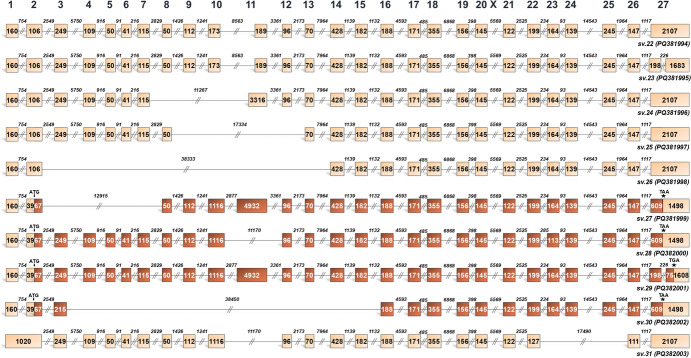
Structural illustration of novel *BRCA2* splice variants expressed only in breast cancer cells (*BRCA2* sv.22 – sv.31). Exons are exhibited as boxes and introns as lines. The numbers inside the boxes and above the lines correspond to the exon and intron lengths, accordingly. Dark brown boxes represent the coding region of the mRNAs that utilize the annotated initiation site of exon 2 and are predicted as protein-coding, while light brown is used to demonstrate non-coding RNAs with PTCs as well as untranslated regions

**Fig. 3:**
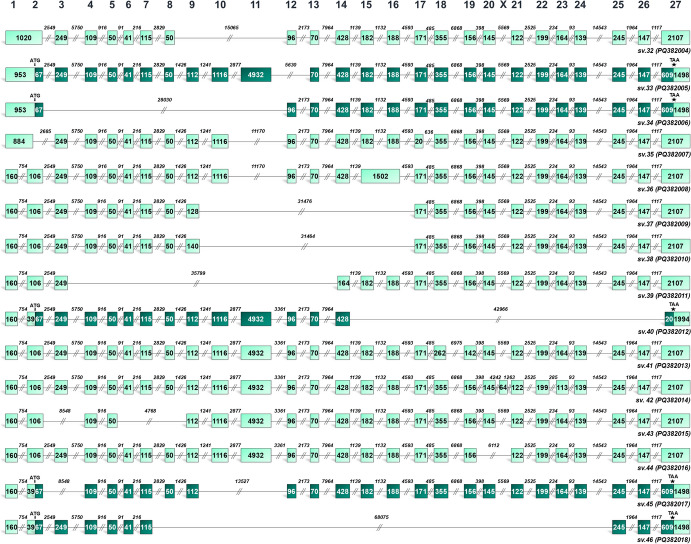
Structural illustration of novel *BRCA2* splice variants expressed only in ovarian cancer cells (*BRCA2* sv.32 – sv.46). Exons are exhibited as boxes and introns as lines. The numbers inside the boxes and above the lines correspond to the exon and intron lengths, accordingly. Dark brown boxes represent the coding region of the mRNAs that utilize the annotated initiation site of exon 2 and are predicted as protein-coding, while light brown is used to demonstrate non-coding RNAs with PTCs as well as untranslated regions

**Fig. 4:**
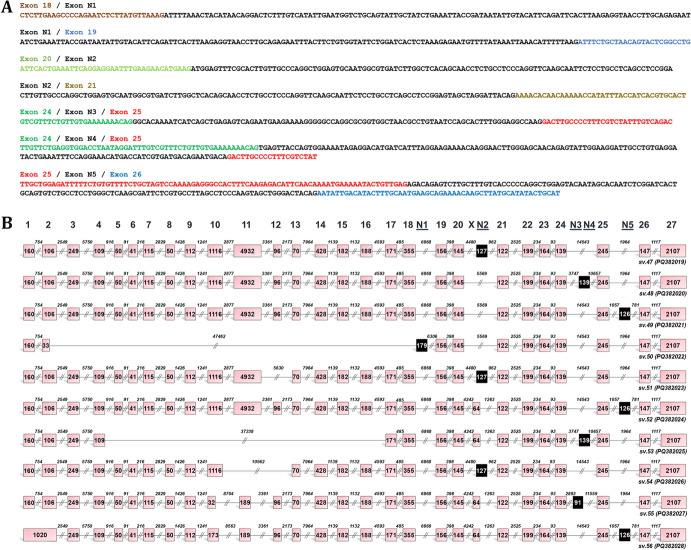
Identification of novel *BRCA2* mRNAs with cryptic exons. **A** Hybrid-seq reads supporting the existence of novel mRNAs bearing cryptic exons, namely N1-N5. For visual purposes, the sequence of each novel exon in the nucleotide sequences is shown in black. **B** Structural illustration of novel *BRCA2* splice variants that contain cryptic exons (*BRCA2* sv.47 – sv.56). Exons are exhibited as boxes and introns as lines. The numbers inside the boxes and above the lines correspond to the exon and intron lengths, accordingly. Black boxes are used to illustrate the described cryptic exons (N1-N5)

The investigation of the *BRCA2* transcriptional landscape in breast and gynecological cancers uncovered 50 previously uncharacterized transcript variants (*BRCA2* sv.7 – sv.56), generated through the gene’s alternative splicing. The sequences of these novel transcripts have been deposited in the GenBank® database under accession numbers PQ381979 – PQ382028, respectively. While many of the identified transcripts exhibit a widespread expression pattern across the cancer types analyzed (Fig. 1), some display cancer-specific expression (Figs. 2 & 3), underscoring potential disease-specific roles. To facilitate characterization, the *BRCA2* transcript variants identified in this study were categorized into two groups: those comprising the annotated exons of the *BRCA2* gene and those containing novel cryptic exons (Fig. 4), the identification of which was a primary objective of the research.

### Identification of novel BRCA2 transcripts containing intact or altered annotated exons

Based on the acquired sequencing data, the newly identified transcript variants *BRCA2* sv.7 – sv.12 resemble the annotated *BRCA2* v.3 since they lack exon 12 but also include additional novel splicing events among the annotated exons (Fig. 1). Following, *BRCA2* sv.13 is characterized by the retention of the intronic sequence that residues between exons 1 and 2, whereas sv.14 has been produced by additional splicing events occurred within exon 11, generating two separate exons, named exon 11a and 11b, which represent distinct regions of the full-length exon 11. Finally, *BRCA2* sv.15 – sv.21 feature a repertoire of novel splice junctions between the annotated exons, generating a collection of diverse novel mRNAs bearing already known exons (Fig. 1).

Interestingly, some of the newly identified transcripts exhibit cancer-specific expression among the tested tumor types. Given the strong predisposition of women with BRCA2 germline alterations to breast and ovarian cancers, particular emphasis was placed on findings derived from these tumor types. Specifically, 10 identified *BRCA2* mRNAs, designated as *BRCA2* sv.22 – sv.31, were exclusively expressed in breast cancer (Fig. 2), while 15 new mRNAs (*BRCA2* sv.32 – sv.46) were identified solely in ovarian cancers (Fig. 3). These findings suggest that *BRCA2* alternative splicing mechanisms vary across different tumor types.

Structural analysis revealed distinct characteristics among the identified transcript variants. In breast cancer cell lines, *BRCA2* sv.22 and sv.23 resemble the annotated *BRCA2* v.1 but include truncated versions of exons 10 and 11, with sv.23 also lacking a portion of exon 27. Similarly, *BRCA2* sv.24 features a truncated sequence of exon 11, which spans 3316 nt, while also lacking annotated exons 8, 9, and 10 (Fig. 2). The subsequent variants, *BRCA2* sv.25 and sv.26, result from extensive exon skipping events, particularly the omission of exons 10 and 11, leading to notably truncated transcripts. Finally, *BRCA2* sv.27 – sv.31 exhibit diverse splicing events, including intron retention, exon skipping as well as the utilization of alternative donor or acceptor sites, producing transcripts that significantly deviate from the annotated mRNAs.

In ovarian cancers, three novel transcripts (*BRCA2* sv.32 – sv.34) retain the first intron of the gene, while BRCA2 sv.35 incorporates a segment of this intron to form an alternative exon 1 of 805 bases. *BRCA2* sv.36 retains the intronic sequence between exons 15 and 16, resulting in an extended exon of 1504 nt. Additionally, *BRCA2* sv.37 – sv.40 are defined by the use of alternative donor and/or acceptor sites, altering the structure of the respective exons (Fig. 3). The remaining ovarian cancer-specific transcripts (*BRCA2* sv.42 – sv.46) arise from multiple cassette exon events involving the annotated exons.

### Cryptic exons contribute to the diversity of the BRCA2 transcriptional landscape

Analysis of the sequencing data led to the identification of five cryptic exons, namely N1 – N5 (Fig. 4). The incorporation of these cryptic exons generates ten novel transcript variants (*BRCA2* sv.47 – sv.56), further enriching the diversity and complexity of the *BRCA2* transcriptional landscape. Transcript variants sv.47 – sv.49 closely resemble *BRCA2* v.1 but include one additional cryptic exon: N2, N4, or N5, respectively (Fig. 4). Similarly, *BRCA2* sv.51 shares sequence similarity with *BRCA2* v.3 but differs by incorporating cryptic exon N2 into its sequence. The novel transcript sv.52 is similar to *BRCA2* v.6, with the addition of cryptic exon N5. Highly truncated variants were also identified, including sv.50 and sv.53, which feature cryptic exons N1 and N4, respectively. Additionally, *BRCA2* sv.54 includes cryptic exon N2 but lacks annotated exons 11 and 12 compared to *BRCA2* v.1. Finally, three additional novel transcripts (*BRCA2* sv.54 – sv.56) were confirmed, each containing one cryptic exon.

### Nanopore sequencing captures the expression levels of BRCA2 transcripts

**Fig. 5:**
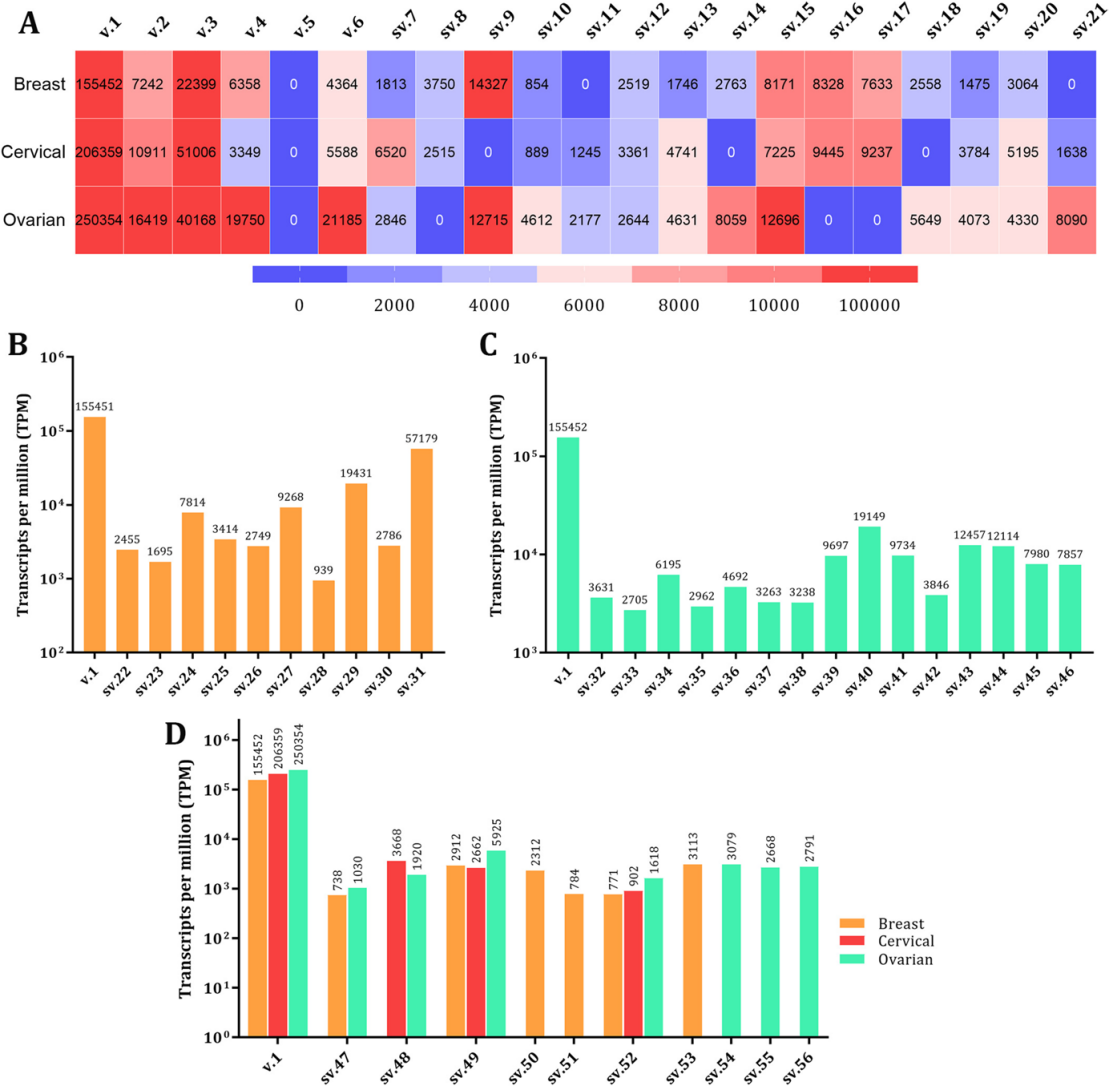
Expression profiling of the novel *BRCA2* splice variants. **A** Heatmap exhibiting the TPM values of the novel *BRCA2* splice variants expressed in more than one cancer type investigated. **B** TPM values of the novel *BRCA2* splice variants expressed in breast cancer cells. **C** TPM values of the novel *BRCA2* splice variants expressed in ovarian cancer cells. **D** TPM values of the novel *BRCA2* splice variants bearing cryptic exons

To provide a comprehensive analysis of the *BRCA2* transcriptional landscape, this study not only identified novel *BRCA2* mRNAs in breast and gynecological cancers (Figs. 2 & 3) but also assessed their expression levels (Fig. 5). Consistent with expectations, the newly identified transcripts were detected at significantly lower levels compared to the primary *BRCA2* v.1. Interestingly, unlike other annotated transcripts, *BRCA2* v.5 was absent in all tested cancer types.

Among the novel transcripts, 15 were expressed in at least two of the cancer types examined, with seven detected across all tested cancers (Fig. 5A). Notably, *BRCA2* sv.9 emerged as one of the most abundant transcripts in breast and ovarian cancers, showing similar expression levels as the annotated *BRCA2* v.2, although it was not detected in cervical cancer. Transcripts sv.15 and sv.49 demonstrated broad expression profiles with significantly higher levels in breast cancer, while sv.16 and sv.17 were prominently expressed in breast and cervical cancers but were absent in ovarian cancer. In contrast, the remaining novel *BRCA2* transcripts exhibited highly restricted expression patterns, being detected in only one cancer type. For instance, sv.29 and sv.31 were among the most abundant transcripts in breast cancer based on TPM values (Fig. 5B), with no expression observed in ovarian or cervical cancers, suggesting potential tissue-specific roles. Conversely, *BRCA2* sv.40 was exclusively expressed in ovarian cancer and was identified as the most abundant novel transcript in this cancer type (Fig. 5C).

Regarding the novel *BRCA2* splice variants bearing cryptic exons, sv. 50 was the only detected mRNA with N1 (179 nt) exon and was found expressed only in breast cancer cells (Fig. 5D). Similarly, cryptic exon N3 (91 nt), which residues between exons 24 and 25, is found to be expressed only in ovarian cancer. On the contrary, cryptic exon N2 (127 nt) exhibits a broader expression pattern since it is present in transcripts derived from both breast and ovarian cancers. Finally, cryptic exons N4 (139 nt) and N5 (126 nt), which are located between the annotated exons 24, 25 and 25, 26, respectively, are expressed in every cancer tested.

### In silico analysis predicts the novel transcripts’ potential functionality

ORF query analysis showed that nineteen of the newly identified transcript variants (*BRCA2* sv.7-sv.9, sv.12-sv.16, sv.19, sv.27-sv.30, sv.33, sv.34, sv.40, sv.45 and sv.46) are predicted to be coding utilizing the annotated start codon for their translation, whereas the remaining transcripts have premature termination codons (PTCs) and, thus are candidates for the NMD pathway (Table 1). Of note, sv.13 and sv.33 are predicted to encode the annotated BRCA2 proteins is.1 and is.3, respectively, since their nucleotide sequence is differentiated from the known transcripts in the 5’ untranslated regions (UTR). The rest of the novel transcripts with translational potential are predicted to encode putative protein isoforms ranging in length from 973 aa to 3386 amino acids.

**Table 1:** List of all the conserved BRCA2 protein domains and their presence/absence in the putative isoforms encoded by the described novel mRNAs

BRCA2 mRNA	Protein length (aa)	Domains						
		PALB-2 BD	RAD51-BD	DBD				C-terminal RAD51-BD
				Helical	OB Fold 1	OB Fold 2	OB Fold 3	
v.1	3418 (is.1)	✓	✓	✓	✓	✓	✓	✓
v.2	3401 (is.2)	✓	✓	✓	✓	–	✓	✓
v.3	3386 (is.3)	✓	✓	✓	✓	✓	✓	✓
v.4	1774 (is.4)	✓	–	✓	✓	✓	✓	✓
v.5	1279 (is.5)	–	–	✓	✓	✓	✓	✓
sv.7	3303	–	✓	✓	✓	✓	✓	✓
sv.8	1742	✓	–	✓	✓	✓	✓	✓
sv.9	3334	✓	✓	✓	–	–	✓	✓
sv.12	3369	✓	✓	✓	✓	-	✓	✓
sv.13	3418	✓	✓	✓	✓	✓	✓	✓
sv.14	2000	✓	–	✓	✓	✓	✓	✓
sv.15	3335	–	✓	✓	✓	✓	✓	✓
sv.16	3366	✓	✓	✓	–	–	✓	✓
sv.19	3366	✓	✓	✓	✓	✓	✓	✓
sv.20	3313	✓	✓	✓	✓	✓	✓	✓
sv.27	3230	–	✓	✓	✓	✓	✓	✓
sv.28	1757	✓	–	✓	✓	–	✓	✓
sv.29	3306	✓	✓	✓	✓	✓	✓	–
sv.30	973	✓	–	–	✓	✓	✓	✓
sv.33	3386	✓	✓	✓	✓	✓	✓	✓
sv.34	1160	-	–	✓	✓	✓	✓	✓
sv.40	2484	✓	✓	–	–	–	–	–
sv.45	1319	–	–	✓	✓	✓	✓	✓
sv.46	543	✓	–	–	–	–	–	✓

In order to evaluate the functionality of these putative proteins, we analyzed their predicted amino acid sequences. Remarkably, the putative BRCA2 isoforms that corresponds to transcripts sv.13, sv.19, sv.20 and sv.33 contain all the characterized domains of the main protein in terms of their amino acid sequence, suggesting strong potential for production and functionality. According to our findings, the putative proteins corresponding to transcripts sv.7, sv.15 and sv.27 are predicted to lack only the PALB-2 binding domain while retaining all the other domains based on amino acid sequence alignment. The isoforms derived from transcripts sv.9, sv.12 and sv.16, are missing one or two oligonucleotide binding (OB) folds within the DNA-binding domain and obtain the rest of the known regions, as exactly the known BRCA2 is.2 does. Furthermore, the isoforms that are predicted to be encoded by transcripts sv.8 and sv.14 are analogous to the annotated is.4, given their lack of the BRC repeats region. Similarly, the putative proteins deriving from sv.34 and sv.45 exhibit the same pattern as is.5, lacking both the PALB-2 binding domain and the region containing the BRC repeats, suggesting common functions among these isoforms.

**Fig. 6:**
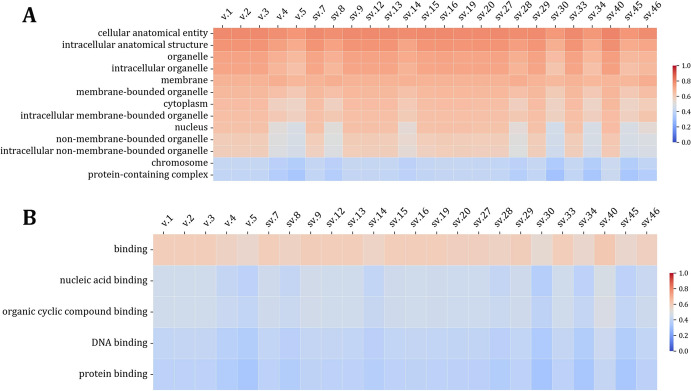
Heatmaps demonstrating the DeepGOWeb scores of the protein-coding annotated and putative novel BRCA2 isoforms regarding their **A** Cellular component and **B** Molecular function

**Fig. 7:**
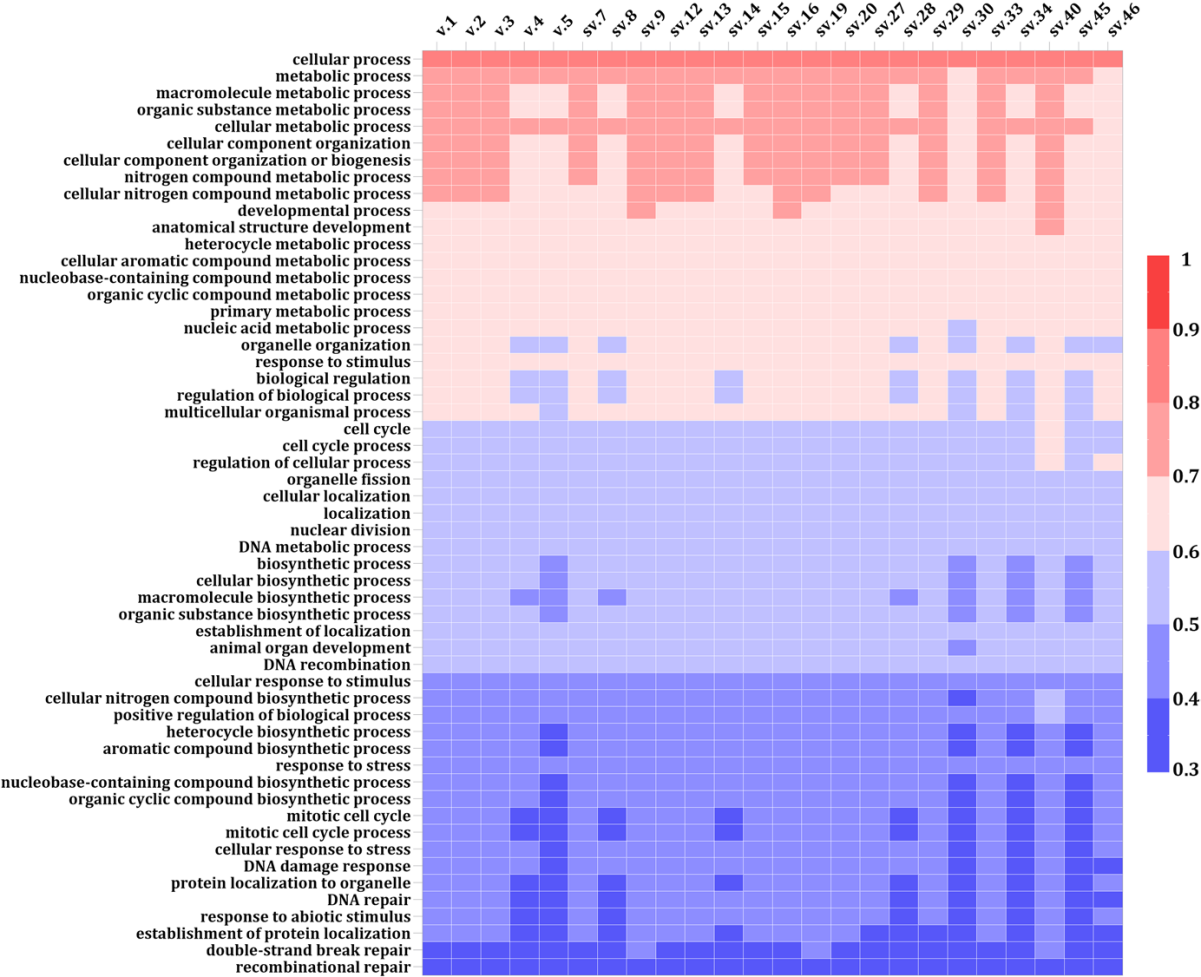
Heatmaps demonstrating the DeepGOWeb scores of the protein-coding annotated and putative novel BRCA2 isoforms regarding their biological process

To further assess the functionality of the predicted BRCA2 proteins, we performed Gene Ontology (GO) analysis. Based on the obtained results, the putative isoforms that correspond to the transcript variants sv.7, sv.9, sv.12, sv.13, sv.15, sv.16, sv.19, sv.20, sv.29 and sv.33 show high similarity to the main BRCA2 isoform in terms of molecular function, suggesting that these isoforms are highly expected to function in the same manner the main BRAC2 does (Fig. 6). Interestingly, the putative isoforms corresponding to the novel transcript sv.40 demonstrates higher scores compared to the main protein in terms of DNA and protein binding, as well as organic cyclic compound binding, a finding that strengthens the hypothesis of its production and functional significance. In contrast, the putative isoforms deriving from transcripts 34 and 45 are characterized by similar GO scores with the BRCA2 is. 5, suggesting that these proteins may share a similar functional profile. Likewise, the probability scores that correspond to the cellular localization of the discussed proteins demonstrate the same pattern as the molecular function, indicating they likely reside in the same cellular compartments as the annotated BRAC2 isoforms (Fig. 6). In addition, all analyzed proteins display high GO scores for cellular processes, exhibiting patterns consistent with BRCA2 proteins across all biological process terms examined (Fig. 7).

## Discussion

Tumor cells present diversity in genetic alterations and gene expression, indicating that every cancer is represented by a subset of unique molecular characteristics (Bianchi et al. 2020). Differential gene expression in cancer holds profound significance in understanding the molecular mechanisms of tumorigenesis and disease progression, while it plays a critical role in cancer subtyping, prognosis, and treatment selection (Estevez-Garcia et al. 2015; Rodriguez-Esteban and Jiang 2017). Advances in high-throughput sequencing have revealed various genes whose expression is altered across cancer types, including breast, ovarian, and cervical cancers. These alterations include gene amplification, deletions, mutations, epigenetic modifications, and aberrant splicing.

Among the genes implicated in cancer pathogenesis, *BRCA2* stands out as a critical element in maintaining genomic integrity and DNA repair mechanisms, making it a significant candidate gene for cancer research (Sadeghi et al. 2020). Its complex structure has hindered comprehensive studies, however, long-read sequencing has overcome this barrier (van Dijk et al. 2018; Athanasopoulou et al. 2021), enabling a deeper investigation into its dysregulation in pathological conditions. In the present study, the implementation of a custom hybrid-seq approach and the generation of highly accurate long reads revealed a fascinating array of expressed *BRCA2* transcripts that comprise cassette exon events as well as cryptic exons and evaluated their expression pattern in gynecological cancers. The discovery of novel *BRCA2* transcript variants highlights the complexity of gene regulation in cancer biology and highlights the potential for targeted therapeutic interventions.

Although the absence of non-cancerous cell lines in the present scientific work might be considered as an omission at first glance, it is not about excluding the normal state from this study. More precisely, even if the preliminary results deriving from the PCR-based assays were encouraging in the case of the cell lines that correspond to breast and gynecological cancers, the expression levels of *BRCA2* in the non-cancerous cell lines that were tested were undetectable, even though a wide panel was investigated (Supplementary Data). These findings come in accordance with a plethora of scientific works which support that *BRCA2* is overexpressed in cancer (Kim et al. 2016; Satyananda et al. 2021), justifying the low expression levels of this gene in normal states. Since the amplification of the *BRCA2* transcripts in non-cancerous cell lines cannot be carried out even though diverse PCR protocols and different polymerases were used for the amplification step, we directed the transcriptional study of this gene in malignancies.

Our investigation into *BRCA2* in breast and gynecological cancers led to the identification of 56 novel *BRCA2* transcript variants. Among these, several transcripts are noteworthy due to their presence in multiple cancer types, suggesting a potential commonality in the molecular landscape across these malignancies. Their existence in multiple cancer types as well as the protein-coding capacity that most of them are predicted to exhibit, underline their potential significance in cellular dysregulation. It should be mentioned that the translational capacity of the identified *BRCA2* mRNAs was investigated in silico based on whether they harbour ORFs utilizing the annotated start codon that exists on the main *BRCA2* mRNA (*BRCA2* v.1). However, the potential utilization of active upstream or downstream AUG codons cannot be excluded. Notably, studies have confirmed that eukaryotic ribosomes can recognize diverse translational start sites, hence translation via alternative ORFs is possible by either reinitiation or leaky scanning (Kochetov et al. 2008; Orr et al. 2020; Dever et al. 2023).

Another major finding of the present study is that some of the described *BRCA2* transcripts demonstrate a very strict expression pattern, being expressed in a specific cancer type. Since *BRCA2* represents a significant candidate in the study of breast and ovarian cancer due to its profound implications in hereditary predisposition, the identification of breast and ovarian cancer-specific *BRCA2* transcripts is of utmost importance. Our sequencing analysis confirmed a total of 13 breast-specific mRNAs with sv.31 being the most abundant mRNA (*BRCA2* sv.22 – sv.31, sv.50, sv.51 and sv.53). Similarly, 18 novel mRNAs were solely detected in ovarian cancer cells (*BRCA2* sv.32 – sv.46, sv.54 – sv.56). Interestingly, 4 novel *BRCA2* mRNAs are expressed only in breast and ovarian cancer and are absent from cervical cancer cell lines, strengthening the association of those cancer types in terms of *BRCA2* hereditary, and potentially suggesting a common mechanism of *BRCA2* dysregulation (Petrucelli et al. 2010; Rebbeck et al. 2015; Wang et al. 2018). Conversely, the identification of cancer-specific transcripts underscores the importance of tailored approaches in understanding and combating each gynecological malignancy. Moreover, these findings may have profound implications for clinical practice, potentially informing personalized treatment strategies based on the unique genetic signatures of individual patients'tumors.

In terms of translational capacity, 19 of the novel *BRCA2* mRNAs harbor ORFs and thus are predicted to coding, while the remainder represent candidates for the NMD pathway (Supek et al. 2021). Notably, sv.13 emerges as a strong candidate for encoding the main BRCA2 isoform, being different from the canonical v.1 only in its 5΄ untranslated region (UTR). This distinction in the 5΄ UTR suggests possible variations in translational regulation, since the secondary structure of this region can influence translation initiation by either facilitating or hindering the scanning of the 43S pre-initiation complex, thereby modulating translational efficiency (Spruill and McDermott 2009). Furthermore, our analysis supports that sv.19, sv.20 and sv.33 are predicted to encode putative BRCA2 isoforms that retain all critical functional domains of the main protein and support comparable role in cellular function (Table 1). The preservation of critical domains in these isoforms strongly suggests that these transcripts could produce functional proteins contributing to BRCA2-related cellular activities.

Interestingly, sv.7, sv.15, and sv.27, which are characterized by a full in-frame exon 3 skipping, are predicted to encode novel BRCA2 proteins similar to the main isoform in terms of length and sequence. However, these isoforms lack PALB2-binding domain, while retaining the rest of the well-characterized BRCA2 domains, indicating a protein missing a critical interaction region. This structural variation may have significant functional implications, as the BRCA2-PALB2 interaction is essential for efficient DNA repair processes. A BRCA2 isoform deficient in PALB2-binding could contribute to a compromised repair mechanism, potentially leading to the accumulation of DNA damage over time and an elevated risk of tumorigenesis. Previous research has confirmed that exon 3 deletion does not affect the translation and stability of the produced protein, but it significantly increases the risk of breast and/or ovarian cancer (Caputo et al. 2018). This finding aligns with our results, suggesting that these coding exon-skipping transcripts could play a role in carcinogenesis by failing to support DNA repair. Accordingly, the putative protein that corresponds to sv.29 is predicted to lack RAD51-binding domain, a deficiency that would impair BRCA2's ability to interact with RAD51 and stabilize RAD51 filaments (Esashi et al. 2005). Since BRCA2-RAD51-interaction is critical for the formation of the “DNA repairing” complex, its absence would hinder homologous recombination and DNA repair. Given the importance of this interaction in the maintenance of genomic integrity, such variations could potentially be significant contributors to oncogenic pathways in BRCA2-associated cancers.

Our in silico analysis also revealed that sv.9, sv.12 and sv.16 are predicted to produce novel BRCA2 isoforms that exhibit high similarity with isoform 2 in terms of amino acid sequence. These isoforms appear to lack one or two OB folds in the DNA-binding domain, yet based on Gene Ontology (GO) scores, they are predicted to maintain functional similarities to isoform 2 (Fig. 6). As such, they are expected to exhibit similar functional roles and cellular localizations. Accordingly, the putative proteins that correspond to sv.34 and sv.45 resemble isoform 5, since they are expected to lack PALB2-binding domain as well as the region that encompass the BRC repeats, a key feature of BRCA2’s interaction with RAD51 and other repair proteins. Based on the prediction scores derived from GO analysis and the sequence similarity these proteins are predicted to have with isoform 2, they are expected to localize in the same cellular components and function in the same way as the latter does.

Lastly, although sv.28, sv.30, sv.40, and sv.46 harbor ORFs, their corresponding putative proteins are unlikely to have functional significance, since they are either predicted to be highly truncated or to contain altered ORFs, resulting in amino acid sequences that differ from those of the annotated proteins. Such truncations and sequence alterations may render these proteins unstable or functionally inactive, further diminishing their potential roles in DNA repair or tumor suppression pathways.

The co-expression of multiple truncated *BRCA2* splice variants alongside the full-length mRNA in cancer cells introduces a layer of regulatory complexity with potentially profound biological consequences. Truncated isoforms that retain partial domain architecture may compete with the full-length BRCA2 protein for essential binding partners such as PALB2 and RAD51, thereby acting in a dominant-negative manner. Isoforms lacking the PALB2-binding domain may still interact with sites of DNA damage but fail to facilitate proper recruitment of RAD51, impeding the formation of functional homologous recombination complexes. Similarly, isoforms lacking the DNA-binding or RAD51-interaction domains may interfere with repair pathways by binding to components without executing downstream functions. Functional interference by truncated isoforms has been well documented in several cancer-associated genes, including *TP53*, where the *Δ133p53* variant inhibits wild-type p53 activity, thus contributing to oncogenesis (Bourdon et al. 2005). In the case of *BCL2L1*, the antagonistic *BCL-XS* and *BCL-XL* variants regulate apoptosis in a splice-dependent manner (Boise et al. 1993). Additional examples include *EGFRvIII* and *p95HER2*, which represent truncated oncogenic isoforms that bypass regulatory control and drive aggressive cancer phenotypes (Scaltriti et al. 2007; Gan et al. 2012). Therefore, the existence of truncated *BRCA2* mRNAs may compromise genome stability and enhance genomic instability—an established hallmark of tumorigenesis. Moreover, altered expression levels among *BRCA2* variants may further influence subcellular localization, protein complex assembly, or checkpoint signaling (Gorodetska et al. 2019). The interplay between full-length and aberrant BRCA2 proteins may contribute to tumor development, progression, or resistance to therapies such as PARP inhibitors. These findings highlight the importance of investigating isoform-specific functions in BRCA2-associated cancers and support the potential of splicing-derived variants as both biomarkers and therapeutic targets.

The identification of the presented novel *BRCA2* mRNAs in breast and gynecological cancers not only enhances our understanding of the molecular landscape of these diseases but also holds promise for advancing precision medicine approaches in the diagnosis and management of these challenging conditions. Understanding the functional impact of these mRNAs on BRCA2 protein function and cellular pathways is crucial for elucidating their roles in tumorigenesis. The identification of these splice variants not only sheds light on the molecular landscape of the investigated cancers but also raises intriguing questions regarding their functional implications. The presence of common mRNAs across multiple cancer types hints at underlying mechanisms or vulnerabilities that transcend individual tumor types, opening avenues for further exploration into potential therapeutic targets or biomarkers with broader applicability.

## Supplementary Information

Below is the link to the electronic supplementary material.

Supplementary file1 (PDF 342 KB)

Supplementary file2 (TIF 1195 KB) Figure 1. Detailed structure of the annotated BRCA2 transcripts (BRCA2 v.1 – v.6). Exons are illustrated as boxes and introns as lines. Numbers inside boxes and above lines denote the length of each exon or intron. Arrows (⇓) are used to represent the ATG site, while asterisks (*) correspond to the position of the termination codon. Dark blue boxes are used to indicate the coding region of each BRCA2 mRNA transcript, while light blue demonstrates the non-coding regions

Supplementary file3 (TIF 3773 KB) Figure 2. Schematic representation of the experimental workflow and bioinformatics pipeline that was performed in the present study

## Data Availability

The datasets related to the present work have been submitted to the Sequence Read Archive (SRA) repository under the BioProject ID PRJNA1269582.
