# Supplementary material for: Decoding the Transcriptional Complexity of the Human BRCA2 DNA Repair Gene Using Hybrid-seq
Source: Biochem Genet. 2025 Jul 10;64(3):3343–66. doi: 10.1007/s10528-025-11180-6 (PMC13186861; doi:10.1007/s10528-025-11180-6)
Supplement: Supplementary file 1 — Supplementary file1 (PDF 342 KB) [file 10528_2025_11180_MOESM1_ESM.pdf]

Supplementary Data

(Sequencing QC reports & Electrophoresis gels)

NanoPlot report

Breast cancer cell lines

Summary statistics

General summary	
Mean read length	3,517.1
Mean read quality	10.8
Median read length	1,035.0
Median read quality	11.5
Number of reads	568,205.0
Read length N50	10,248.0
STDEV read length	4,091.6
Total bases	1,998,460,948.0
Number, percentage and megabases of reads above quality cutoffs	
>Q5	568205 (100.0%) 1998.5Mb
>Q7	568205 (100.0%) 1998.5Mb
>Q10	447890 (78.8%) 1720.3Mb
>Q12	234060 (41.2%) 1157.0Mb
>Q15	15479 (2.7%) 97.2Mb
Top 5 highest mean basecall quality scores and their read lengths	
1	18.3 (323)
2	18.2 (542)
3	18.2 (307)
4	18.1 (396)
5	18.0 (397)
Top 5 longest reads and their mean basecall quality score	
1	50338 (9.0)
2	30662 (15.5)
3	29359 (11.4)
4	26938 (12.6)
5	26687 (10.6)

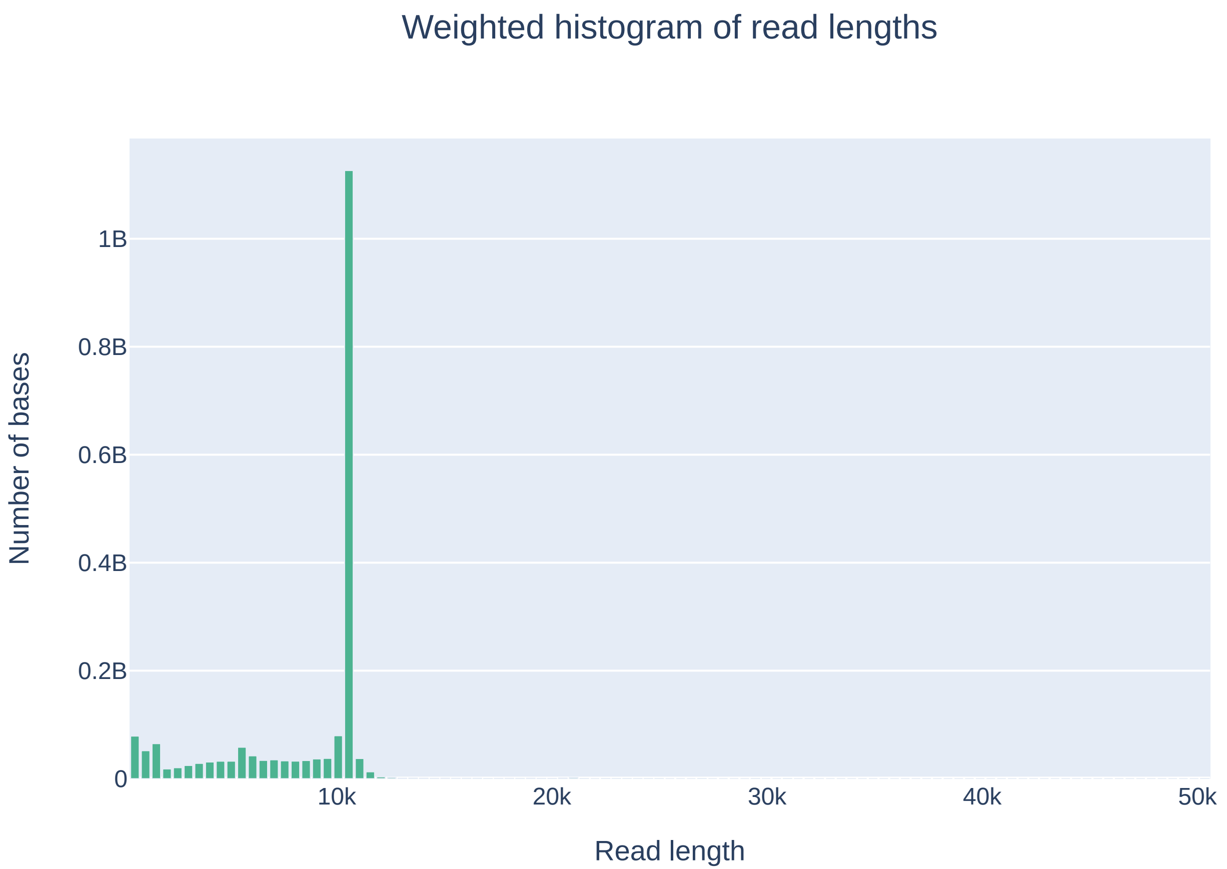

Loading [MathJax]/extensions/MathMenu.js

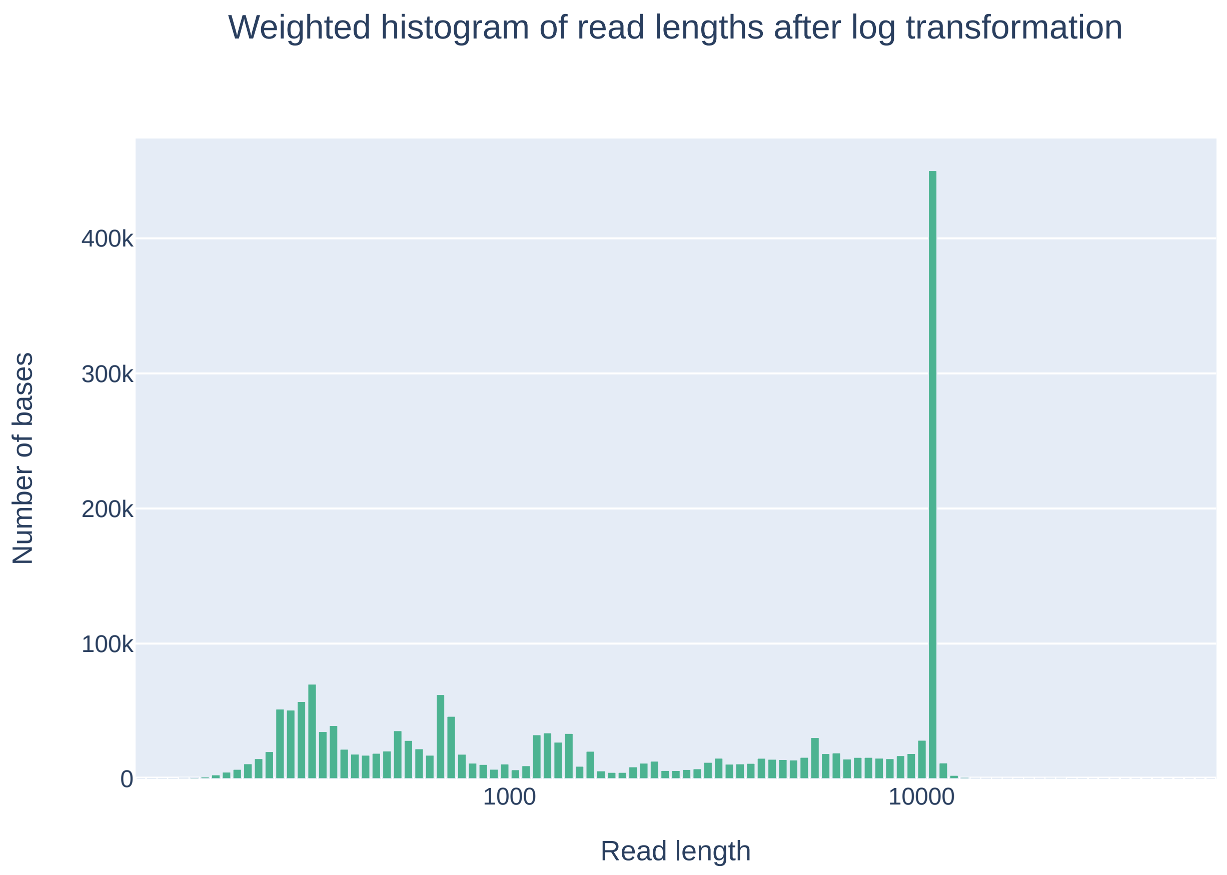

Loading [MathJax]/extensions/MathMenu.js

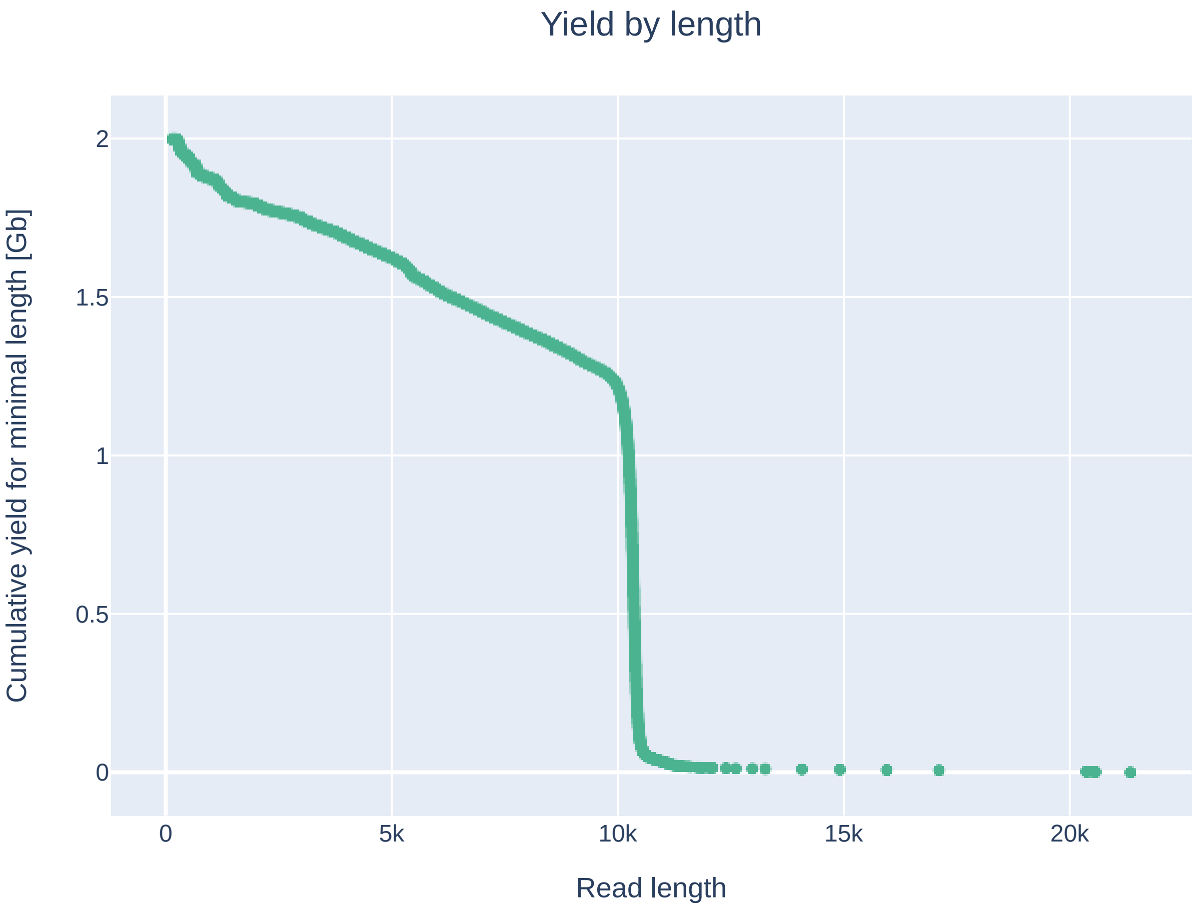

Loading [MathJax]/extensions/MathMenu.js

NanoPlot report

Cervical cancer cell lines

Summary statistics

General summary	
Mean read length	4,749.3
Mean read quality	10.5
Median read length	2,627.0
Median read quality	11.2
Number of reads	163,424.0
Read length N50	10,260.0
STDEV read length	4,324.8
Total bases	776,149,089.0
Number, percentage and megabases of reads above quality cutoffs	
>Q5	163424 (100.0%) 776.1Mb
>Q7	163424 (100.0%) 776.1Mb
>Q10	115075 (70.4%) 634.5Mb
>Q12	63524 (38.9%) 416.2Mb
>Q15	5120 (3.1%) 33.3Mb
Top 5 highest mean basecall quality scores and their read lengths	
1	21.1 (173)
2	19.3 (305)
3	19.2 (271)
4	18.6 (609)
5	18.5 (411)
Top 5 longest reads and their mean basecall quality score	
1	30442 (9.9)
2	28436 (10.2)
3	28407 (8.9)
4	28212 (8.9)
5	27282 (12.3)

Weighted histogram of read lengths

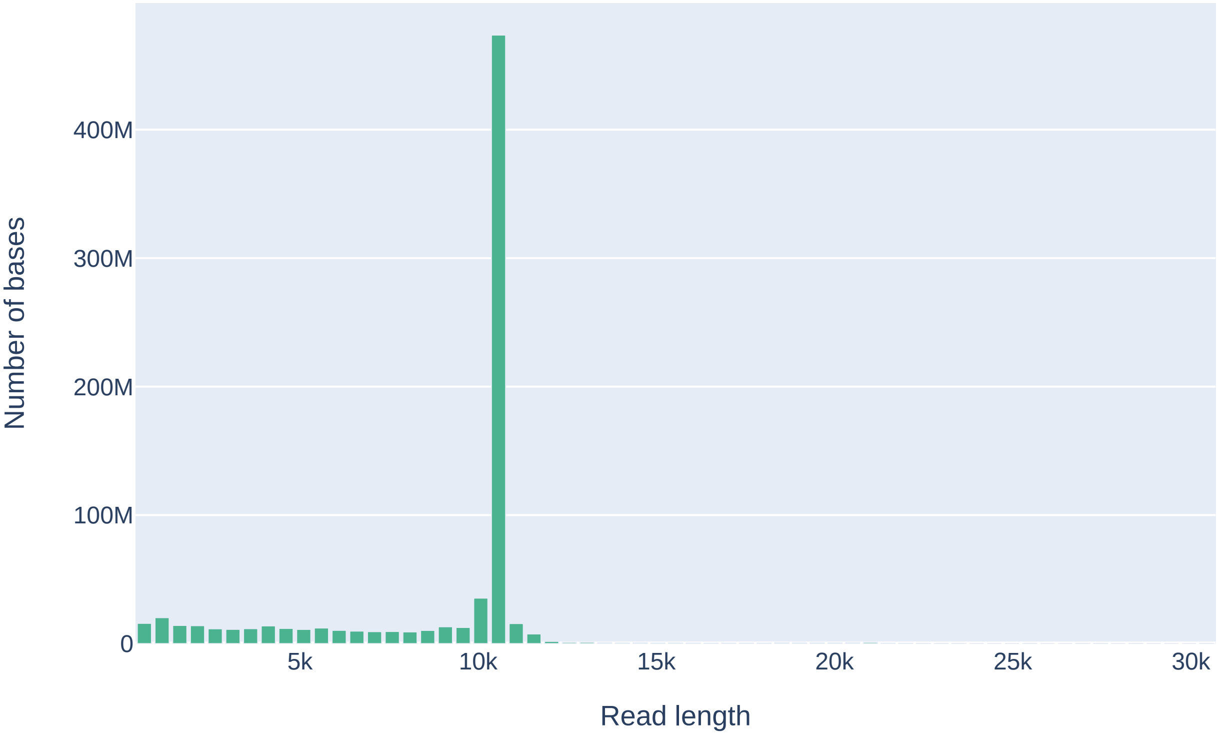

Loading [MathJax]/extensions/MathMenu.js

Weighted histogram of read lengths after log transformation

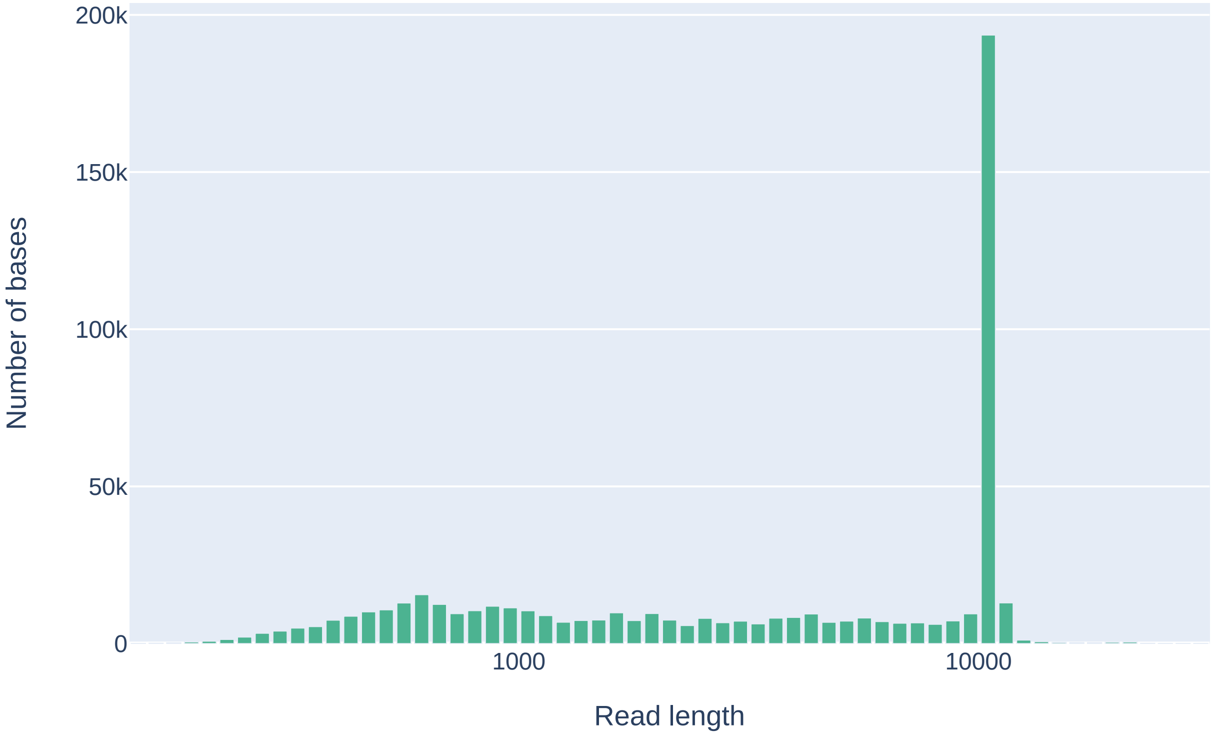

Loading [MathJax]/extensions/MathMenu.js

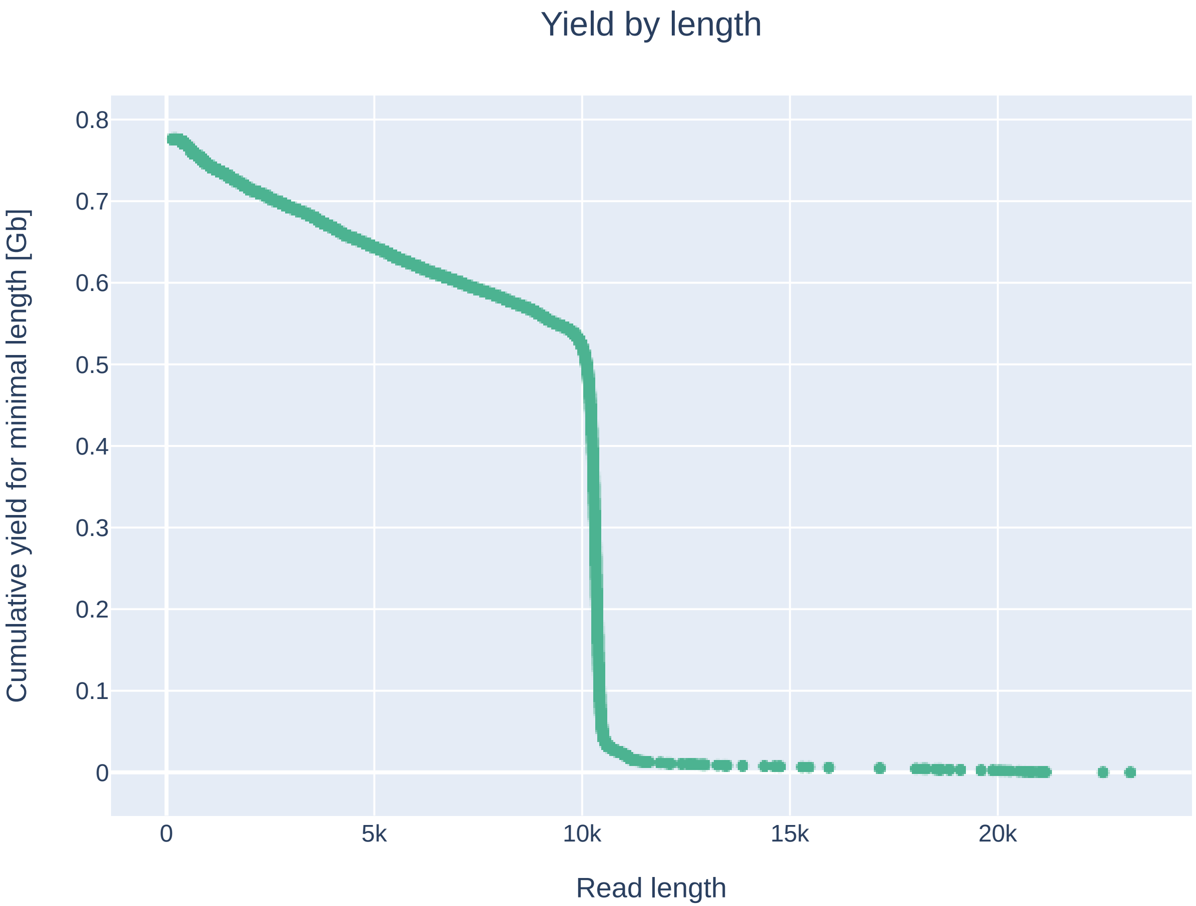

Loading [MathJax]/extensions/MathMenu.js

NanoPlot report

Ovarian cancer cell lines

Summary statistics

**General summary**	
**Mean read length**	2,303.0
**Mean read quality**	10.4
**Median read length**	464.0
**Median read quality**	11.1
**Number of reads**	504,892.0
**Read length N50**	10,173.0
**STDEV read length**	3,474.4
**Total bases**	1,162,745,574.0
**Number, percentage and megabases of reads above quality cutoffs**	
**>Q5**	504892 (100.0%) 1162.7Mb
**>Q7**	504892 (100.0%) 1162.7Mb
**>Q10**	372477 (73.8%) 965.5Mb
**>Q12**	161335 (32.0%) 617.8Mb
**>Q15**	9364 (1.9%) 49.8Mb
**Top 5 highest mean basecall quality scores and their read lengths**	
**1**	19.8 (367)
**2**	19.2 (299)
**3**	18.8 (344)
**4**	18.7 (404)
**5**	18.5 (6336)
**Top 5 longest reads and their mean basecall quality score**	
**1**	25438 (13.2)
**2**	23900 (10.3)
**3**	22498 (8.6)
**4**	22470 (12.8)
**5**	22158 (13.7)

Weighted histogram of read lengths

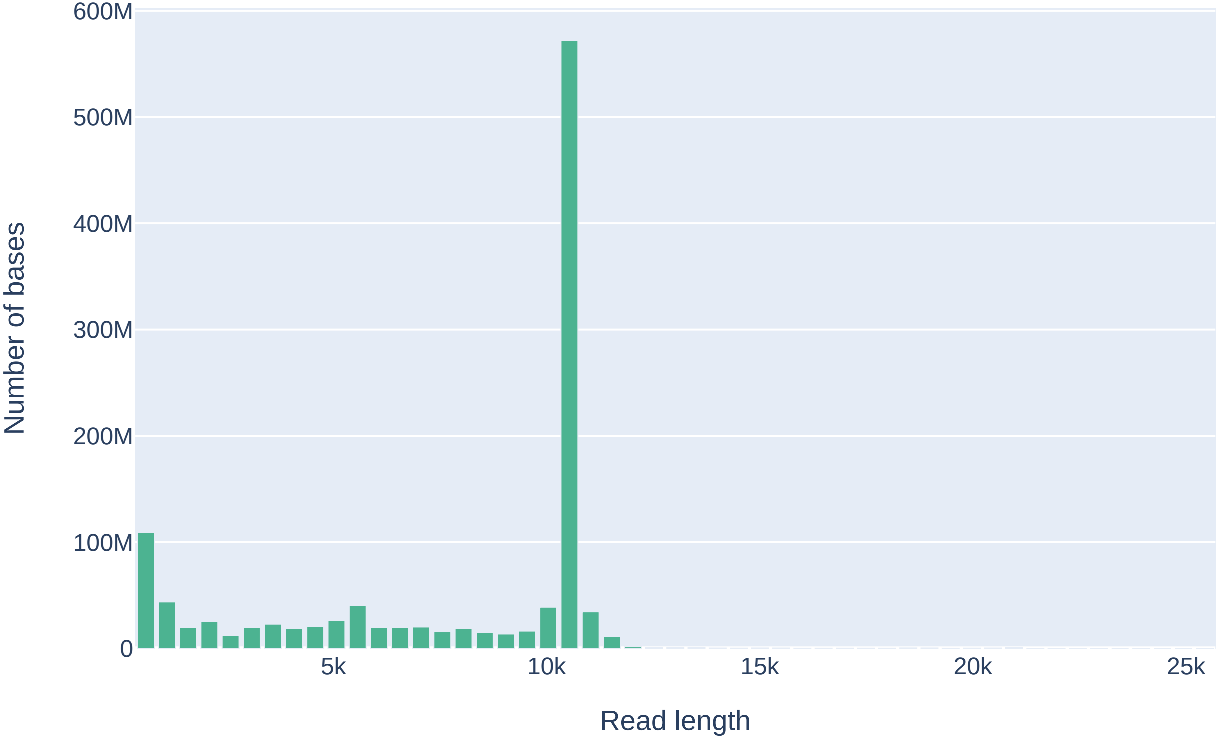

Loading [MathJax]/extensions/MathMenu.js

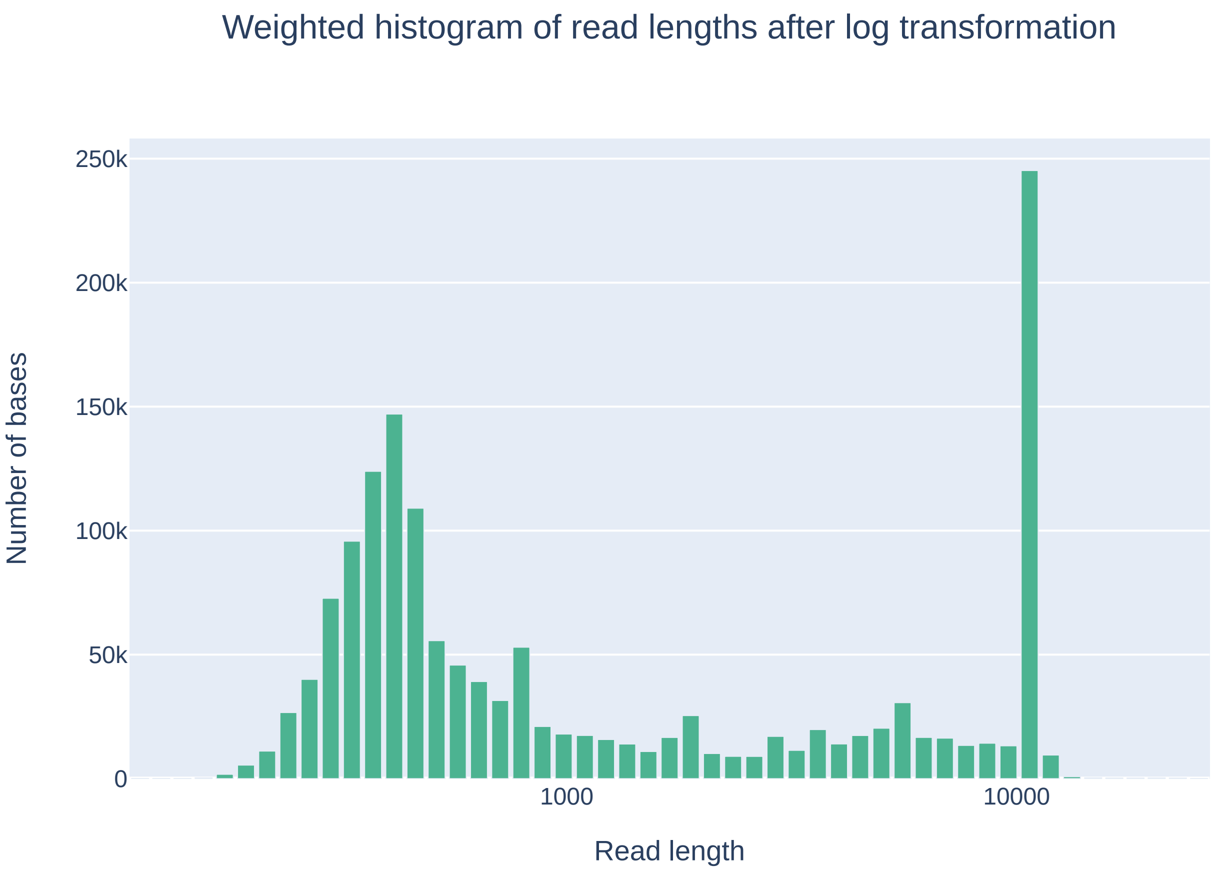

Loading [MathJax]/extensions/MathMenu.js

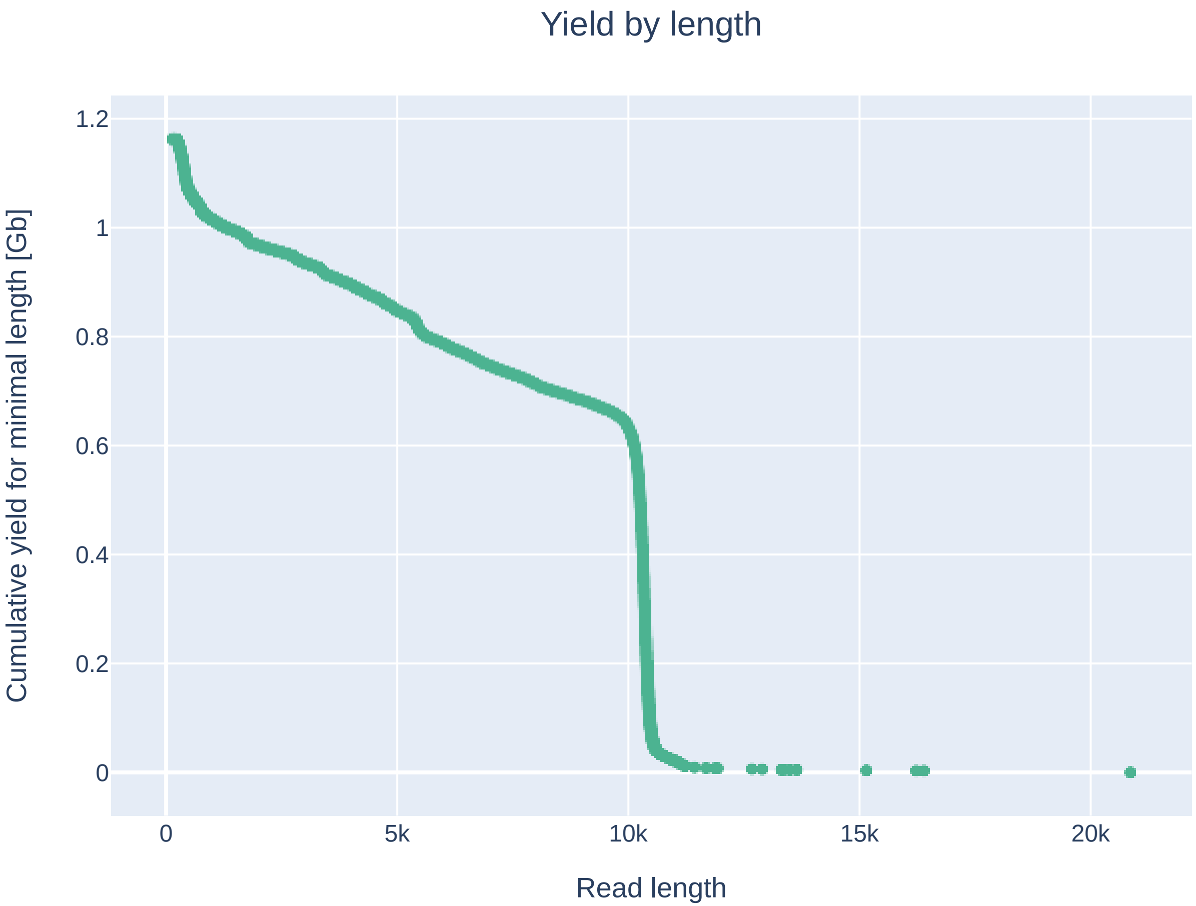

Loading [MathJax]/extensions/MathMenu.js

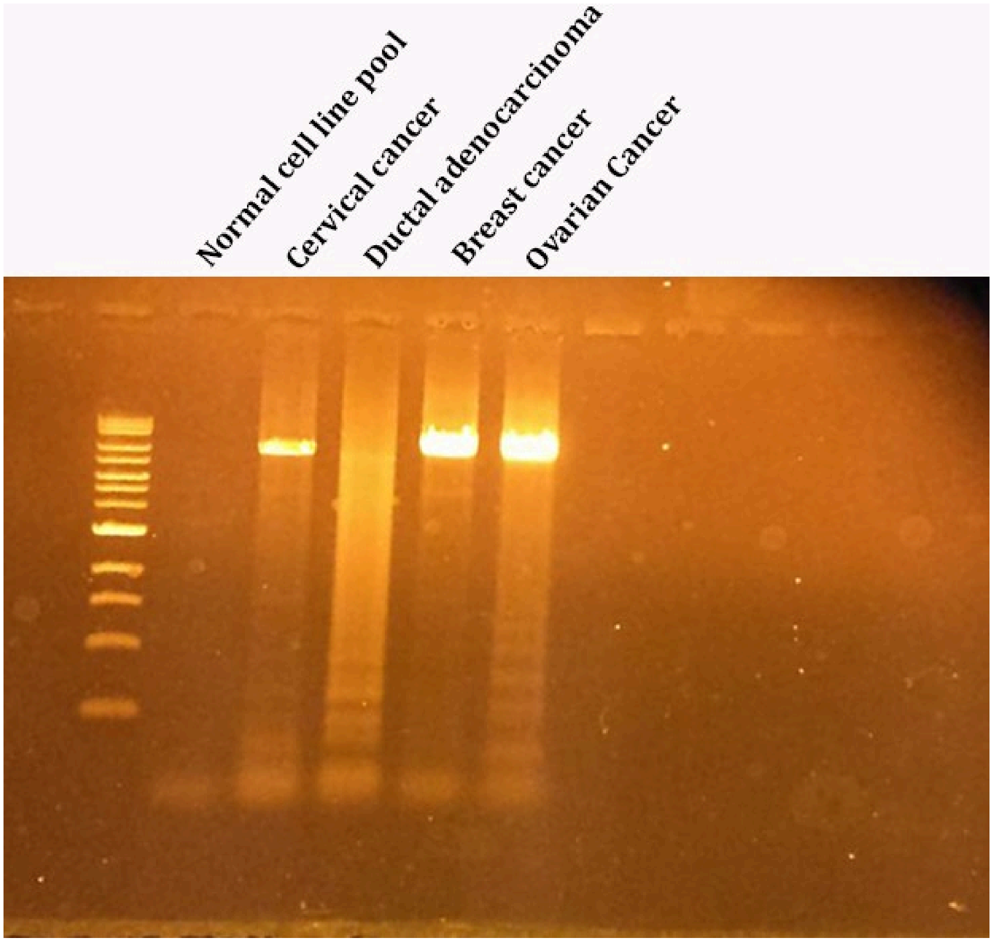
Normal cell line pool
Cervical cancer
Ductal adenocarcinoma
Breast cancer
Ovarian Cancer

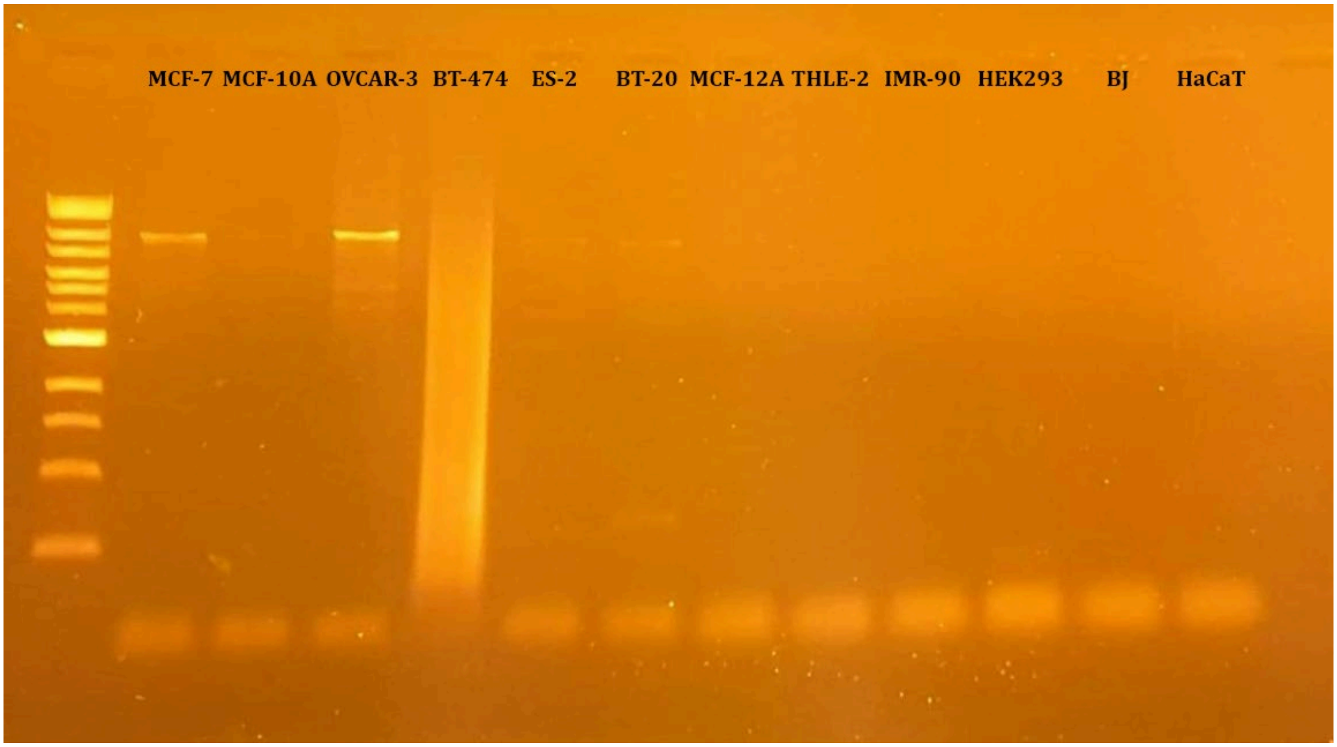
MCF-7
MCF-10A
OVCAR-3
BT-474
ES-2
BT-20
MCF-12A
THLE-2
IMR-90
HEK293
BJ
HaCaT
