# Supplementary figures and images for: Decoding the Transcriptional Complexity of the Human BRCA2 DNA Repair Gene Using Hybrid-seq
Source: Biochem Genet. 2025 Jul 10;64(3):3343–66. doi: 10.1007/s10528-025-11180-6 (PMC13186861; doi:10.1007/s10528-025-11180-6)

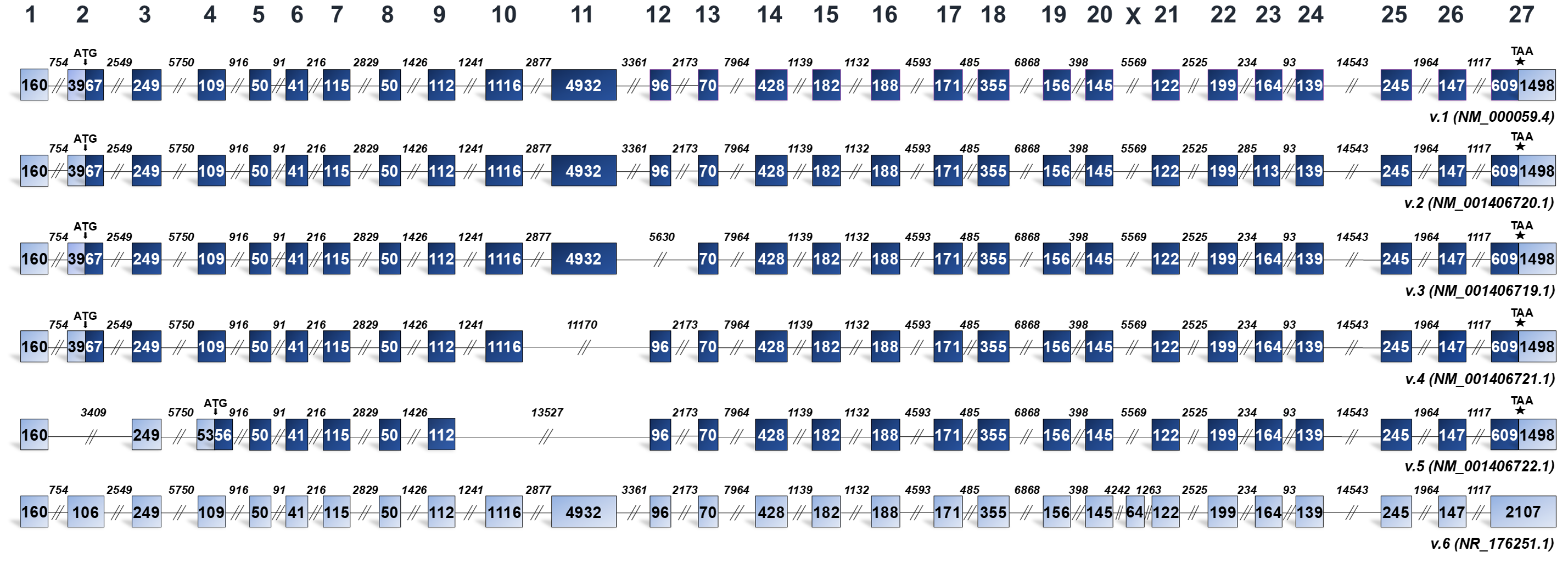

Supplement: Supplementary file 2 — Supplementary file2 (TIF 1195 KB) Figure 1. Detailed structure of the annotated BRCA2 transcripts (BRCA2 v.1 – v.6). Exons are illustrated as boxes and introns as lines. Numbers inside boxes and above lines denote the length of each exon or intron. Arrows (⇓) are used to represent the ATG site, while asterisks (*) correspond to the position of the termination codon. Dark blue boxes are used to indicate the coding region of each BRCA2 mRNA transcript, while light blue demonstrates the non-coding regions [file 10528_2025_11180_MOESM2_ESM.tif]

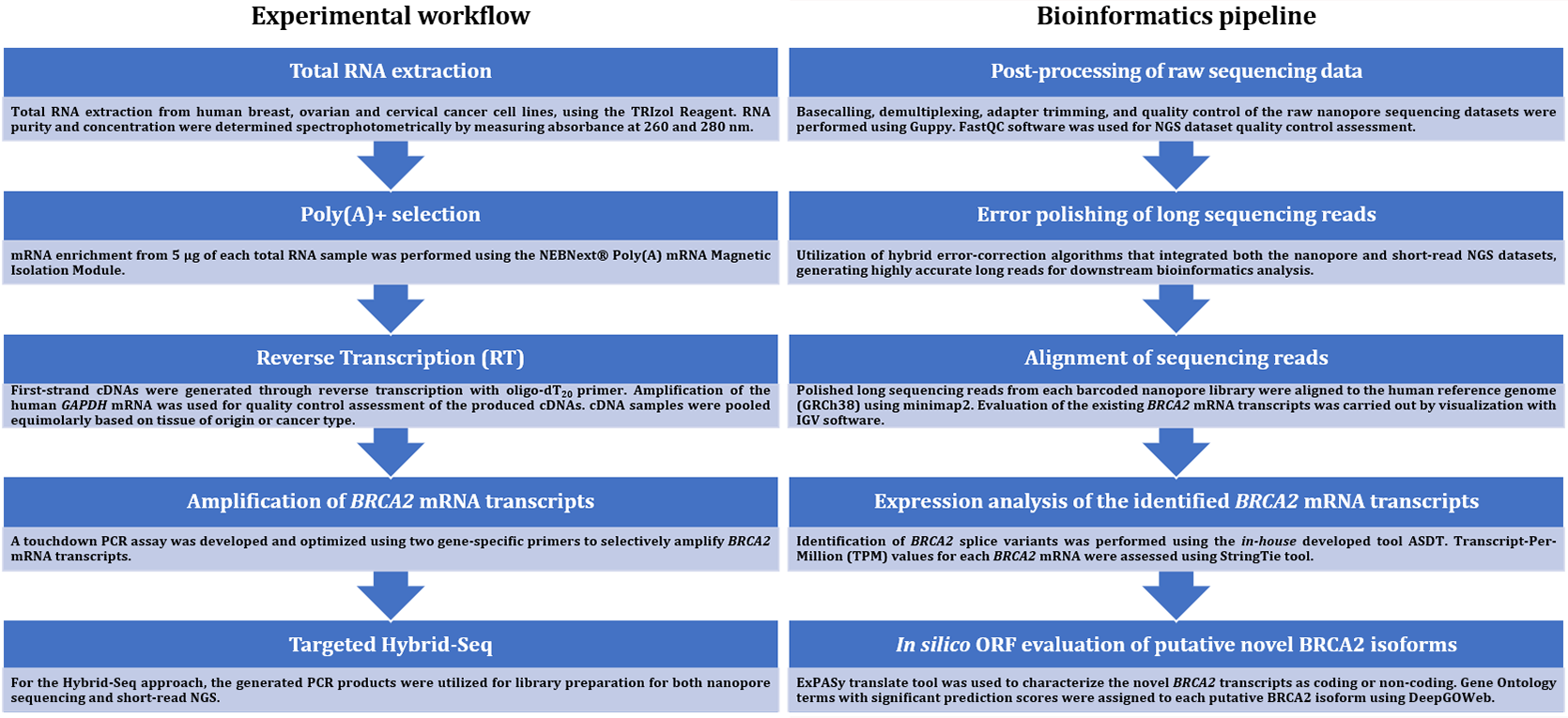

Supplement: Supplementary file 3 — Supplementary file3 (TIF 3773 KB) Figure 2. Schematic representation of the experimental workflow and bioinformatics pipeline that was performed in the present study [file 10528_2025_11180_MOESM3_ESM.tif]
